# Association of Continuity of Care With Outcomes in US Veterans With Inflammatory Bowel Disease

**DOI:** 10.1001/jamanetworkopen.2020.15899

**Published:** 2020-09-04

**Authors:** Shirley Cohen-Mekelburg, Sameer D. Saini, Sarah L. Krein, Timothy P. Hofer, Beth I. Wallace, John M. Hollingsworth, Julie P. W. Bynum, Wyndy Wiitala, Jennifer Burns, Peter D. R. Higgins, Akbar K. Waljee

**Affiliations:** 1 Division of Gastroenterology & Hepatology, Department of Internal Medicine, University of Michigan, Ann Arbor; 2 Center for Clinical Management Research, Ann Arbor Veterans Affairs Health System, Ann Arbor, Michigan; 3 Department of Internal Medicine, University of Michigan, Ann Arbor; 4 Division of Rheumatology, Department of Internal Medicine, University of Michigan, Ann Arbor; 5 Department of Urology, University of Michigan, Ann Arbor; 6 Division of Geriatrics, Department of Internal Medicine, University of Michigan, Ann Arbor

## Abstract

**Importance:**

Health care fragmentation is associated with inefficiency and worse outcomes. Continuity of care (COC) models were developed to address fragmentation.

**Objective:**

To examine COC and selected outcomes in US veterans with inflammatory bowel disease (IBD).

**Design, Setting, and Participants:**

This retrospective cohort study used the Veterans Health Administration (VHA) Corporate Data Warehouse to identify veterans with IBD who received care in the VHA health care system between January 1, 2002, and December 31, 2014. Included patients were veterans with IBD who had a primary care physician and at least 4 outpatient visits with key physicians (gastroenterologist, primary care physician, and surgeon) within the first year after an index IBD encounter. Data were analyzed from November 2018 to May 2020.

**Exposures:**

Care continuity was measured with the Bice-Boxerman COC index to define care density and dispersion within year 1 after the initial presentation.

**Main Outcomes and Measures:**

A Cox proportional hazards regression model was used to quantify the association between a low level of COC in year 1 (defined as ≤0.25 on a 0 to 1 scale) and subsequent IBD-related outcomes in years 2 and 3 (outpatient flare, hospitalization, and surgical intervention).

**Results:**

Among the 20 079 veterans with IBD who met the inclusion criteria, 18 632 were men (92.8%) and the median (interquartile range [IQR]) age was 59 (48-66) years. In the first year of follow-up, substantial variation in the dispersion of care was observed (median [IQR] COC, 0.24 [0.13-0.46]). In a Cox proportional hazards regression model, a low level of COC was associated with a higher likelihood of outpatient flares that required corticosteroids (adjusted hazard ratio [aHR], 1.11; 95% CI, 1.01-1.22), hospitalizations (aHR, 1.25; 95% CI, 1.06-1.47), and surgical interventions (aHR, 1.72; 95% CI, 1.43-2.07).

**Conclusions and Relevance:**

Results of this cohort study showed a wide variation in dispersion of IBD care and an association between a lower level of COC and active IBD and worse outcomes. The findings suggest that investigating the barriers to COC in integrated systems that have invested in care coordination is key to not only better understanding COC but also identifying opportunities to improve care fragmentation.

## Key Points

**Question:**

Is a low level of continuity of care among patients with inflammatory bowel disease (IBD) associated with poor IBD-related outcomes?

**Findings:**

In this cohort study of 20 079 US veterans with IBD receiving care in the Veterans Health Administration system, a low level of continuity of care was associated with a higher likelihood of flares that required corticosteroid treatment, hospitalization, and surgical intervention.

**Meaning:**

This cohort study found that continuity of care varied in patients with IBD, even in an integrated system with systematically enhanced care coordination; this finding suggests the need to improve care coordination for patients with complex chronic conditions.

## Teaser

This cohort study examines the fragmentation and dispersion of primary and specialist care along with care outcomes among patients with inflammatory bowel disease treated in the Veterans Health Administration health care system.

## Introduction

Health care in the United States is marked by substantial fragmentation, with patients pursuing and receiving care from multiple clinicians, often at different institutions.^1^ Fragmented care has been associated with poor chronic disease outcomes, higher health care use, duplication in testing, and increased costs of care.^2,3,4^ In the past decade, various programs, often concentrated on primary care, have been implemented to reduce fragmentation and promote high-value coordinated care. A primary care medical home model, known as the Patient Aligned Care Team (PACT), was implemented in more than 800 clinics in the Veterans Health Administration (VHA) health care system.^5^ In the PACT model, a designated primary care physician (PCP) leads a clinical care team who sees the patient regularly and coordinates care as both the point of first entry to the health system and as the principal source of referrals to specialists and other health care practitioners. However, these efforts are less relevant to patients with complex chronic medical conditions that require comanagement with specialists.

Inflammatory bowel disease (IBD) is one example of such a chronic medical condition that requires longitudinal comanagement by both a specialist (gastroenterologist) and a PCP. An estimated 3 million Americans live with IBD—a high-expenditure low-prevalence disease—and IBD care is estimated to have a direct cost of $14.6 billion and an indirect cost of $31.6 billion annually.^6,7^ Therefore, promotion of effective and efficient IBD care is paramount. High-quality care for IBD includes not only disease-specific management of symptoms but also disease-specific preventive care, such as immunizations and cancer screening, to prevent associated adverse outcomes.^8,9^ Identifying which physician is responsible for managing each aspect of care requires some degree of coordination and makes patients with IBD vulnerable to care fragmentation.

In this cohort study, we quantified care continuity (a measure of fragmentation and a key aspect of coordination) and described its association with outcomes for patients with IBD in the VHA system. The primary objective was to examine continuity of care (COC) among veterans with IBD and the association between low levels of COC and selected IBD-related outcomes (ie, outpatient corticosteroid-treated flares, hospitalizations, and surgical interventions).^10,11^

## Methods

Using the VHA Corporate Data Warehouse, an administrative database of clinical and other data for all veterans who receive care within the VHA system across the US, we identified patients with IBD with 1 or more outpatient encounters between January 1, 2002, and December 31, 2014. The cohort study protocol was reviewed and approved by the VA Ann Arbor Health System Institutional Review Board, which waived the requirement for informed consent. We followed the Strengthening the Reporting of Observational Studies in Epidemiology (STROBE) reporting guideline.

A previously validated algorithm based on a combination of inpatient and outpatient *International Classification of Diseases, Ninth Revision, Clinical Modification* (*ICD-9-CM*) codes for Crohn disease (555.x) and ulcerative colitis (556.x)^12^ was applied to ascertain IBD status. Patients were eligible for inclusion if they had 2 or more of these *ICD-9-CM* codes during at least 2 clinical encounters during the study period, with at least 1 encounter being an outpatient visit according to previously validated algorithms.^12,13,14^ To be included, patients had to have at least 1 visit with a PCP during the study period. For study purposes, the date of the first encounter with either an *ICD-9-CM* code of 555.x or 556.x was considered the IBD index date. Patient follow-up was carried out for the first 3 years after the index date, and follow-up data over time were analyzed. We focused on patient encounters with gastroenterologists, PCPs, and surgeons, the 3 major clinical physicians in IBD care. Patients with fewer than 4 encounters with any of these key physicians in the first year of follow-up were excluded to reduce the bias of limited observations on the COC index.

We identified all gastroenterologist, PCP, and general surgical (including colorectal) outpatient visits within the VHA system. Care continuity was calculated using the Bice-Boxerman COC index over this 1-year period. This index is the sum of the difference between the squared product of the number of visits with a particular clinician and the total number of visits over the study period that is then divided by the product of the total number of visits over the study period and the total number of visits minus 1.^15^ The COC index is a measure of care dispersion and density and reflects the extent to which a patient’s medical visits are connected with a distinct physician.^4,15,16^ As originally conceptualized, the COC index was designed to measure fragmentation of care across treating teams comprising a PCP and any specialists to whom the PCP referred a patient.^16^ Given the difficulty of establishing the origin of referrals that drive patient encounters, most subsequent studies have used the COC index simply to describe fragmentation across all physicians, regardless of referral origin, or fragmentation within episodes of care or across PCPs alone.^3,4,16^ Trainees and physician extenders in the VHA system practice under an attending physician and thus were not considered as individual clinicians.

Furthermore, the Bice-Boxerman COC index does not measure direct communication or comanagement between clinicians. A COC index score of 0 demonstrates complete discontinuity, when each visit is with a different physician, whereas an index score of 1 is consistent with perfect continuity, when all visits are with the same physician. Given that patients with IBD require both a gastroenterologist and a PCP at a minimum, the expected COC index for a patient with IBD is approximately 0.5 if the visits are evenly divided between the PCP and the gastroenterologist.

### Statistical Analysis

The Bice-Boxerman COC index for year 1 was treated as the primary independent variable. Given the skewed distribution of the COC variable and for ease of interpretation, we dichotomized the COC variable, categorizing a low level of COC as a COC index of 0.25 or lower on a scale of 0 to 1.^17,18,19,20^ The prespecified outcomes included outpatient flares requiring corticosteroids, hospitalization, and surgical intervention that occurred in years 2 or 3 of the study period; year 1 outcomes were excluded to control for any potential impact of the outcome on the COC. Outpatient flares were identified on the basis of filled outpatient prescriptions for corticosteroids using a previously described algorithm.^10^ Hospitalizations were inpatient stays associated with an *ICD-9-CM* code for IBD, and corticosteroids were corticosteroid prescriptions filled during the admission.^10^ All of the *ICD-9-CM* codes and variables used to identify these outcomes, including IBD-related surgical procedures, are presented in eTable 1 in the Supplement.

A multivariable Cox proportional hazards regression model was used to explore time to each outcome, censoring patients at the time each outcome occurred or at the end of the 3-year follow-up period. This model controlled a priori for age, race, Charlson Comorbidity Index (score range of each comorbidity: 1-6, with higher scores indicating a greater likelihood of 1-year mortality), outpatient flare in year 1, sex, and IBD type, in addition to adjusting SEs for clustering at the facility level to account for differences between hospitals that cannot easily be measured. History of an outpatient flare in year 1 was included as a factor associated with disease severity. The association between the Bice-Boxerman COC index for year 1 and the available covariates was initially assessed using χ^2^ or Fisher exact test for categorical covariates. An unpaired, 2-tailed *t* test or Wilcoxon rank sum test was used for continuous covariates, as appropriate. Subsequently, a multivariable logistic regression model was used to identify factors most strongly associated with COC. We performed a sensitivity analysis excluding patients who died during the 3-year follow-up period so that all patients would have the same exposure time.

### Sensitivity Analyses

Many past studies used a COC index cutoff of 0.25 to define a low level of COC, but this cutoff is an arbitrary threshold. Although dichotomizing COC allows for ease of interpretation, to account for any patterns lost in clustering values, we performed a sensitivity analysis that considered year 1 COC level as a continuous variable. To better understand fragmentation within specialties, we examined the association between outcomes and COC in a single physician type. Two additional sensitivity analyses explored other measures of disease severity—year 1 hospitalizations and baseline use of immunomodulator or biological agent. Two subgroup analyses were performed that included only patients with an identified VHA gastroenterologist and excluded patients who underwent surgical intervention given the anticipated higher likelihood of capturing low levels of COC among patients with a VHA gastroenterologist and high levels of COC among patients who underwent a surgical procedure. A final set of subgroup analyses explored the association between COC and outcomes when excluding patients with severe disease who may be more likely to have a higher visit frequency and to receive urgent care from different physicians.

All statistical analyses were performed with Stata/MP, version 14.0 (StataCorp LLC). Data were analyzed from November 2018 to May 2020.

## Results

**Figure 1.**
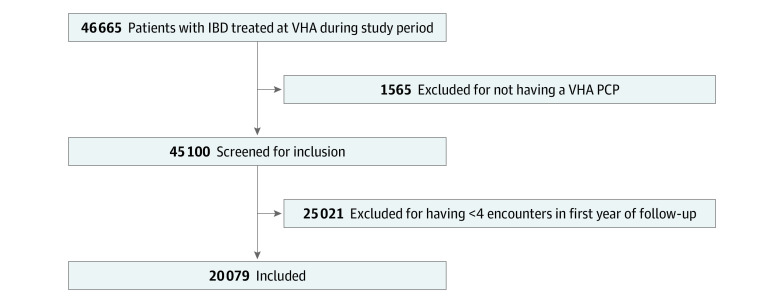
Study Population IBD indicates inflammatory bowel disease; PCP, primary care physician; and VHA, Veterans Health Administration.

Among the 20 079 veterans with IBD who met the inclusion criteria (Figure 1), 18 632 were men (92.8%) and 1447 were women (7.2%), 15 346 were White (76.4%) individuals, and the median (interquartile range [IQR]) age was 59 (48-66) years (Table 1). This cohort had a mean (SD) Charlson Comorbidity Index score of 1.32 (1.83). Although this cohort represents a subpopulation of veterans with IBD who received comprehensive care in the VHA system, to address selection bias, we compared their baseline characteristics with the characteristics of those excluded and found similarities (eTable 2 in the Supplement). However, data on excluded patients likely underrepresent the medication use for severe disease and outcomes, both of which may be managed outside of the VHA system. Over years 2 and 3 of follow-up, 4658 patients (23.2%) had an outpatient flare, 2068 (10.3%) required hospitalization, and 943 (4.7%) required a surgical procedure.

**Table 1.** Baseline Demographic Characteristics and Continuity of Care^a^

Characteristic	Overall proportion, No. (%)	COC, median (IQR)
**Patient-level factors**		
Total No. of veterans	20 079	0.24 (0.13-0.46)
Age, y		
≤50	5521 (28.0)	0.19 (0.10-0.36)
51-65	8328 (42.3)	0.25 (0.14-0.47)
>65	5862 (29.7)	0.29 (0.16-0.50)
Sex		
Male	18 632 (92.8)	0.24 (0.13-0.41)
Female	1447 (7.2)	0.20 (0.13-0.41)
Race		
White	15 346 (76.4)	0.24 (0.14-0.47)
Non-White	4733 (23.6)	0.21 (0.13-0.41)
IBD type		
Crohn disease	7789 (38.8)	0.21 (0.13-0.42)
Ulcerative colitis	10 967 (54.6)	0.26 (0.14-0.48)
Indeterminate colitis	1323 (6.6)	0.20 (0.13-0.40)
CCI score, mean (SD)		
0-2	16 261 (81.0)	0.22 (0.13-0.67)
>2	3818 (19.0)	0.27 (0.14-0.48)
Region		
Northeast	4480 (22.3)	0.22 (0.12-0.44)
Southeast	4871 (24.3)	0.20 (0.12-0.42)
Continental	701 (35.1)	0.25 (0.14-0.47)
Pacific	3677 (18.3)	0.25 (0.14-0.46)
**Baseline factors**		
Corticosteroid-treated flares	2363 (11.8)	0.20 (0.11-0.35)
No	17 716 (88.2)	0.24 (0.13-0.42)
Hospitalizations	882 (4.4)	0.17 (0.10-0.26)
No	19 197 (95.6)	0.24 (0.13-0.42)
Immunomodulator or biological agent use	4041 (20.1)	0.18 (0.11-0.30)
No medication use	16 038 (79.9)	0.25 (0.14-0.44)
Immunomodulator agent	3276 (16.3)	NA
Biological agent	1874 (9.3)	NA
Infliximab	1003 (5.0)	NA
Adalimumab	824 (4.1)	NA
Certolizumab pegol	47 (0.2)	NA
**Facility-level factors**		
Facility complexity level		
Highest	9326 (46.5)	0.20 (0.11-0.40)
High	4321 (21.5)	0.20 (0.13-0.40)
Mid-high	3532 (17.6)	0.29 (0.17-0.48)
Medium	1390 (6.9)	0.33 (0.20-0.50)
Low	1506 (7.5)	0.33 (0.19-0.60)
Rural	6196 (30.9)	0.27 (0.14-0.48)
Not rural	13 883 (69.1)	0.22 (0.13-0.43)

Abbreviations: CCI, Charlson Comorbidity Index (score range of each comorbidity: 1-6, with higher scores indicating a greater likelihood of 1-year mortality); COC, continuity of care; IBD, inflammatory bowel disease; IQR, interquartile range; NA, not applicable.

^a^ Values are specific to year 1.

A total of 11 103 patients (55.3%) visited more than 1 PCP, whereas 8975 (44.7%) had a single PCP. In contrast, 4176 patients (20.8%) saw 1 gastroenterologist, 11 786 (58.7%) saw more than 1 gastroenterologist, and 4116 (20.5%) saw no VHA gastroenterologists during the study period. Patients had a median (IQR) of 7 (4-10) PCP visits and a median (IQR) of 4 (2-7) gastroenterologist visits. Of the 4928 patients (24.5%) who visited a surgeon, 1974 (40.1%) saw 1 surgeon and 2954 (59.9%) saw more than 1 surgeon over the study period.

**Figure 2.**
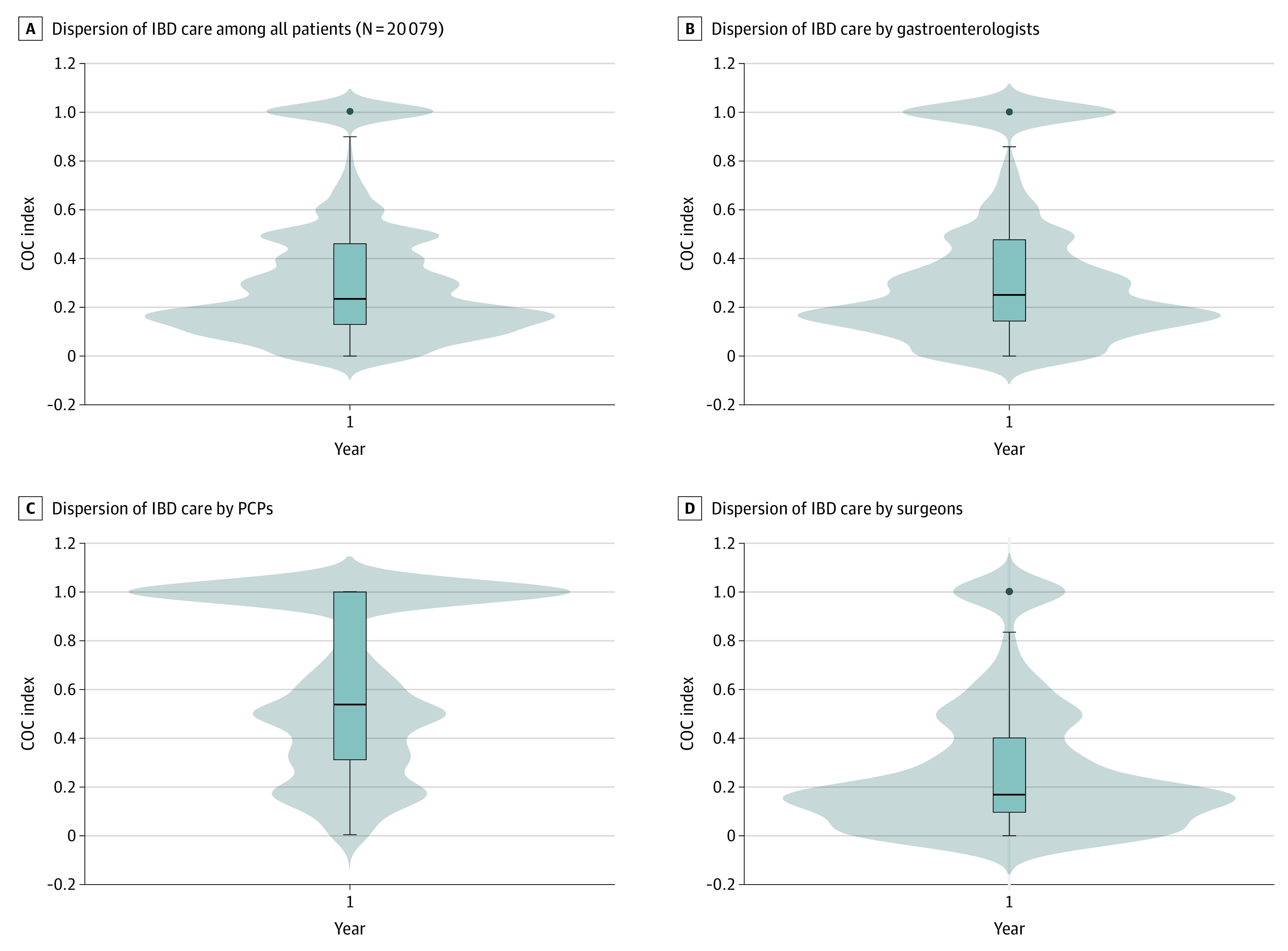
Distributions of Bice-Boxerman Continuity of Care (COC) Index A box plot is superimposed on a violin plot to show the full distribution of COC values. From the top down, the horizontal bars represent the maximum, third quartile, median, first quartile, and minimum values. The dots represent outliers. IBD indicates inflammatory bowel disease; PCP, primary care physician.

We observed substantial variability in the dispersion of IBD care among physicians in a bimodal distribution (Figure 2). The median (IQR) COC level in year 1 of follow-up was 0.24 (0.13-0.46) (Table 1). With respect to IBD-specific events, 2291 of 4654 outpatient flares (49.2%), 683 of 2077 hospitalizations (32.9%), and 390 of 951 surgical procedures (41.0%) occurred in year 2 or year 3 of follow-up. Substantial variability in COC by facility was also evident. After controlling for facility-level factors, such as facility complexity and rurality, a lower level of COC was associated with younger age (3361 [32.5%]), baseline use of immunomodulator or biological agent (2515 [23.8%]), Crohn disease (5498 [52.1%]) or indeterminate colitis (762 [7.2%]), and hospitalizations (603 [5.7%]) in year 1 (Table 2). In a sensitivity analysis excluding patients who died during the 3-year follow-up period, similar associations persisted (eTable 3 in the Supplement).

**Table 2.** Associations Between Continuity of Care and Patient- and Facility-Level Factors ^a^

Characteristic	COC, No. (%)		Adjusted OR (95% CI)
	≤0.25 Index	>0.25 Index	
**Patient**-**level factors**			
No. of veterans (%)	10 557 (52.6)	9522 (47.4)	NA
Age, y			
≤50	3361 (32.5)	2160 (23.1)	1.66 (1.54-1.81)
51-65	4277 (41.3)	4051 (43.3)	1.19 (1.11-1.28)
>65	2710 (26.2)	3152 (33.7)	1 [Reference]
Sex			
Male	9755 (92.4)	8877 (93.2)	1 [Reference]
Female	802 (7.6)	645 (6.8)	0.95 (0.85-1.07)
Race			
White	7976 (75.6)	7370 (77.4)	0.97 (0.91-1.04)
Non-White	2581 (24.5)	2152 (22.6)	1 [Reference]
IBD type			
Crohn disease	5498 (52.1)	5469 (57.4)	1.13 (1.06-1.20)
Ulcerative colitis	4297 (40.7)	3492 (36.7)	1 [Reference]
Indeterminate colitis	762 (7.2)	561 (5.9)	1.17 (1.04-1.32)
CCI, mean (SD)			
0-2	8655 (82.0)	7606 (79.9)	1 [Reference]
>2	1902 (18.0)	1916 (20.1)	1.02 (0.95-1.10)
Region			
Northeast	2408 (22.8)	2072 (21.8)	0.95 (0.86-1.03)
Southeast	2665 (25.2)	2206 (23.2)	0.90 (0.82-0.99)
Continental	3587 (34.0)	3464 (36.4)	0.80 (0.74-0.87)
Pacific	1897 (18.0)	1780 (18.9)	1 [Reference]
**Baseline factors**			
Corticosteroid-treated flares	1347 (12.8)	1016 (10.7)	0.95 (0.86-1.05)
No	9210 (87.2)	8506 (89.3)	1 [Reference]
Hospitalizations	603 (5.7)	279 (2.9)	1.62 (1.38-1.90)
No	9954 (94.3)	9243 (97.1)	1 [Reference]
Immunomodulator or biological agent use	2515 (23.8)	1526 (16.0)	1.42 (1.31-1.53)
No	8042 (76.2)	7996 (84.0)	1 [Reference]
**Facility-level factors**			
Facility complexity level			
Highest	5481 (51.9)	3845 (40.4)	1 [Reference]
High	2414 (22.9)	1907 (20.0)	0.86 (0.80-0.93)
Mid-high	1649 (15.6)	1883 (19.8)	0.64 (0.59-0.69)
Medium	490 (4.6)	900 (9.5)	0.36 (0.32-0.41)
Low	522 (5.0)	984 (10.3)	0.36 (0.32-0.41)
Rural	3087 (29.2)	3109 (32.7)	0.98 (0.92-1.05)
Not rural	7470 (70.8)	6413 (67.4)	1 [Reference]

Abbreviations: CCI, Charlson Comorbidity Index (score range of each comorbidity: 1-6, with higher scores indicating a greater likelihood of 1-year mortality); COC, continuity of care; IBD, inflammatory bowel disease; NA, not applicable; OR, odds ratio.

^a^ Values are specific to year 1.

Using a Cox proportional hazards regression model, a low level of COC within year 1 was associated with a higher likelihood of outpatient flares requiring corticosteroids (adjusted hazard ratio [aHR], 1.11; 95% CI, 1.01-1.22), hospitalizations (aHR, 1.25; 95% CI, 1.06-1.47), and surgical interventions (aHR, 1.72; 95% CI, 1.43-2.07) in subsequent years after controlling for a priori covariates and adjusting SEs for clustering at the facility level (Table 3). These associations did not change when limiting the cohort to patients who were alive at the end of the follow-up period (Table 3). For example, the aHR was 1.11 (95% CI, 1.01-1.23) for outpatient flares requiring corticosteroids, 1.29 (95% CI, 1.09-1.52) for hospitalizations, and 1.70 (95% CI, 1.39-2.09) for surgical interventions. Similar results were also evident when considering COC for year 1 as a continuous variable. An improvement in COC index score by 0.1 was associated with a lower likelihood of an outpatient flare (aHR, 0.69; 95% CI, 0.58-0.82), hospitalization (aHR, 0.57; 95% CI, 0.41-0.79), and surgical intervention (aHR, 0.25; 95% CI, 0.16-0.38).

**Table 3.** Association Between a Low Level of Continuity of Care and Inflammatory Bowel Disease–Related Outcomes

Outcome	Adjusted HR (95% CI)
Low level of total COC (N = 20 079)^a^	
Outpatient flares	1.11 (1.01-1.22)
Hospitalizations	1.25 (1.06-1.47)
Surgical interventions	1.72 (1.43-2.07)
Low level of gastroenterologist-specific COC (n = 6841)	
Outpatient flares	0.76 (0.66-0.88)
Hospitalizations	0.94 (0.74-1.20)
Surgical interventions	0.78 (0.61-1.01)
Low level of primary care physician–specific COC (n = 10 292)	
Outpatient flares	1.05 (0.91-1.21)
Hospitalizations	0.88 (0.69-1.14)
Surgical interventions	0.99 (0.68-1.46)
Low level of total COC (extended definition of IBD severity) (n = 20 079)^b^	
Outpatient flares	0.96 (0.88-1.05)
Hospitalizations	1.19 (1.01-1.40)
Surgical interventions	1.52 (1.26-1.83)
Subgroup of patients with VHA gastroenterologist (n = 15 965)	
Outpatient flares	0.95 (0.87-1.05)
Hospitalizations	1.05 (0.88-1.24)
Surgical interventions	1.48 (1.22-1.80)
Subgroup of patients excluding deaths (n = 18 825)	
Outpatient flares	1.11 (1.01-1.23)
Hospitalizations	1.29 (1.09-1.52)
Surgical interventions	1.70 (1.39-2.09)
Subgroup of patients with nonsevere disease (n = 17 716)^c^	
Outpatient flares	1.11 (1.01-1.22)
Hospitalizations	1.23 (1.03-1.47)
Surgical interventions	1.77 (1.42-2.20)
Subgroup of nonsurgical patients (n = 15 151)	
Outpatient flares	1.07 (0.96-1.18)
Hospitalizations	1.37 (1.10-1.71)
Surgical interventions	NA

Abbreviations: IBD, inflammatory bowel disease; COC, continuity of care; HR, hazard ratio; VHA, Veterans Health Administration.

^a^ COC index score of ≤0.25.

^b^ Includes IBD hospitalizations in year 1 and use of immunomodulator or biological agents as covariates.

^c^ Severity defined by corticosteroid-treated flares in year 1.

When considering varying measures of disease severity (year 1 hospitalizations and baseline use of immunomodulator or biological agent) or in subgroup analyses of patients with nonsevere IBD and nonsurgical patients, the association between a low level of COC and outcomes persisted (Table 3). Among patients with nonsevere IBD, the aHR was 1.11 (95% CI, 1.01-1.22) for outpatient flares requiring corticosteroids, 1.23 (95% CI, 1.03-1.47) for hospitalizations, and 1.77 (95% CI, 1.42-2.20) for surgical interventions. Among nonsurgical patients, the aHR was 1.37 (95% CI, 1.10-1.71) for hospitalizations. In an additional subgroup analysis limited to patients with a VHA gastroenterologist, a lower level of COC continued to be associated with surgical interventions (aHR, 1.48; 95% CI, 1.22-1.80) but not outpatient flares (aHR, 0.95; 95% CI, 0.87-1.05) or hospitalizations (aHR, 1.05; 95% CI, 0.88-1.24). Physician-specific COC varied, with a median (IQR) COC index score in year 1 of 0.25 (0.14-0.478) for gastroenterologists, 0.54 (0.31-1.00) for PCPs, and 0.17 (0.10-0.40) for surgeons (Figure 2).

When considering physician-specific COC, the association between a low level of COC and outcomes no longer existed (Table 3). For low gastroenterologist-specific COC, the aHR was 0.76 (95% CI, 0.66-0.88) for outpatient flares requiring corticosteroids, 0.94 (95% CI, 0.74-1.20) for hospitalizations, and 0.78 (95% CI, 0.61-1.01) for surgical interventions. For low PCP-specific COC, the aHR was 1.05 (95% CI, 0.91-1.21) for outpatient flares requiring corticosteroids, 0.88 (95% CI, 0.69-1.14) for hospitalizations, and 0.99 (95% CI, 0.68-1.46) for surgical interventions.

## Discussion

The infrastructure and alignment of an integrated health care delivery system, such as the VHA, may provide the ideal environment for care coordination. Yet, even in this setting, COC, an important aspect of care coordination, varies among patients with IBD, and a low level of COC is associated with worse outcomes. The overall level of COC in the population of veterans with IBD has been lower than the typical COC level reported for patients with congestive heart failure, chronic obstructive pulmonary disease, or diabetes (as close to 0.50), although such studies were generally performed outside of the VHA or using a non-VHA data set.^4^ The level of COC among patients with IBD in the present VHA cohort was also lower than the values described in previous studies of veterans in the VHA system, including a study of VHA-Medicare dual enrollees who were especially prone to fragmented care because of their ability to seek care both inside and outside of the VHA system.^21^

The difference in COC among patients with IBD vs patients without IBD is likely multifactorial and may be associated with confusion about physician accountability and lack of focus on coordination in IBD multidisciplinary care. Patients with IBD require care by PCPs, gastroenterologists, and surgeons, but the delineation of responsibility by physician is often unclear. Integration of care of patients with IBD can be suboptimal because of specialty care access issues, a lack of communication between PCPs and specialists, and insufficient knowledge of IBD-specific quality measures.

Despite a national emphasis on promoting coordination of care for patients with certain chronic conditions, such as congestive heart failure, chronic obstructive pulmonary disease, and diabetes, fewer resources and incentives have been invested in other chronic conditions such as IBD, which are not targeted in this manner.^4,22^ Open-access clinics, in which patients may see any number of available physicians rather than being assigned to a specific physician, are common practice in both primary and specialty care. This practice likely also contributes to the lack of COC demonstrated in this study.

Although fragmentation has been associated with inappropriate care, there is always a component of appropriate fragmentation present that is associated with second opinions or transfers to a center of excellence, which may be necessary to provide high-quality care.^23,24^ Appropriate fragmentation is particularly important in discussions of the management of severe and complex conditions, such as IBD, in which regional expertise may be limited to specific physicians or institutions. To account for the potential implication of appropriate discontinuity for patients with IBD with more severe disease, we included measures of disease severity as a covariate in the multivariable model.

Care continuity is essential to improving care delivery for chronic disease and needs to be addressed in the management of IBD.^25^ In response, the VHA PACT model, which has constructed team-based care clinics with primary care as the hub, emerged. Identification of an accountable clinician of care and establishment of resources to promote timely access are key aspects of this transformation.^26,27,28^ For chronic, complex conditions such as IBD, care coordination may be improved through the use of specialty care medical homes.^29,30,31^ However, although the medical home model has been implemented in the primary care settings of the VHA system, evidence that supports this approach for IBD care in the VHA is lacking.^5,32^ Furthermore, wide acceptability and feasibility of a specialty care medical home model could be difficult to achieve given limited financial resources and substantial variability in incentives among specialists.

This study took the first step in better understanding COC in a population of patients with chronic gastrointestinal disease. Investigating current barriers to COC in a system that has invested in care coordination is key to understanding and eventually improving COC and to addressing care fragmentation in IBD. Future studies should incorporate a measure for team-based care, provided by groups of clinicians, to understand and improve coordination in IBD care.^33^

### Limitations

This study has limitations. We used administrative data, such as billing codes, to identify patients and limited data on IBD extent, duration, and behavior. However, this IBD study cohort was well established, with a known sensitivity and specificity for using the defined *ICD-9-CM* codes. We focused on a population of veterans with IBD, which may not be completely generalizable to a non-VHA population. However, the VHA provided us with an opportunity to study continuity of care in a system that has invested heavily in promoting coordination and quality of chronic disease care. To reduce bias caused by a low number of visits, we used strict criteria to calculate COC, which required 4 encounters. Therefore, we were only able to include patients with IBD with calculable year 1 COC indices, which may limit generalizability to patients with high health care use. However, our study question focused on patients with IBD who received comprehensive care within the VHA system that may either be fragmented or continuous rather than on patients who interacted with the VHA system infrequently and for whom the association of care with outcomes may be different. We also explored the differences when considering only nonsurgical patients with IBD, although the implication of multidisciplinary care for care coordination requires further exploration. In addition, this study identified only physicians, although the PACT model promotes team-based care. As such, we were limited in our ability to fully describe the impact of nonphysician care teams.

Disease severity is associated with the frequency with which patients receive care, the pursuit of multidisciplinary care, and clinical outcomes. In both the primary analysis and sensitivity analyses, we controlled for disease severity and overall comorbidity among other factors, but unmeasurable confounders may persist. Furthermore, we were unable to account for care outside of the VHA system for patients who obtained care on a fee basis from community clinicians or who had dual enrollment with Medicare or Medicaid. Analyzing the care provided in the VHA system and in the community is important, but first the current state of IBD care within the VHA must be evaluated to serve as a basis for future studies.

## Conclusions

This cohort study found that the level of COC for patients with IBD was low, even in an integrated system, such as the VHA, that engages in systematic efforts to enhance care continuity and coordination. A lower level of COC was also associated with worse outcomes. Providing PCPs and specialists the resources to help comanage complex chronic conditions is essential. Neither continuity nor coordination was simple so as to capture using a single index. To our knowledge, this study was the first to examine COC in a population of veterans with chronic gastrointestinal disease. Investigating the current barriers in a system that has invested heavily in care coordination is key to understanding and eventually improving COC as well as addressing care fragmentation for patients with IBD. Future studies should incorporate a measure for team-based care.

## Notes

Supplement. **eTable 1.** Component Variables Associated With Outcomes **eTable 2.** Characteristics of Excluded Patients **eTable 3.** Sensitivity Analysis of Associations Excluding Deaths
