# Supplementary material for: Association of Continuity of Care With Outcomes in US Veterans With Inflammatory Bowel Disease
Source: JAMA Netw Open. 2020 Sep 4;3(9):e2015899. doi: 10.1001/jamanetworkopen.2020.15899 (PMC7489806; doi:10.1001/jamanetworkopen.2020.15899)
Supplement: Supplement. — eTable 1. Component Variables Associated With Outcomes eTable 2. Characteristics of Excluded Patients eTable 3. Sensitivity Analysis of Associations Excluding Deaths [file jamanetwopen-e2015899-s001.pdf]

Supplementary Online Content

Cohen-Mekelburg S, Saini SD, Krein SL, et al. Association of continuity of care with outcomes in US veterans with inflammatory bowel disease. *JAMA Netw Open*. 2020;3(9):e2015899. doi:10.1001/jamanetworkopen.2020.15899

eTable 1. Component Variables Associated With Outcomes

eTable 2. Characteristics of Excluded Patients

eTable 3. Sensitivity Analysis of Associations Excluding Deaths

This supplementary material has been provided by the authors to give readers additional information about their work.

©2020 Cohen-Mekelburg S et al. *JAMA Network Open*

eTable 1. Component Variables Associated With Outcomes

Types of systemic corticosteroids:	
prednisone, methylprednisolone, hydrocortisone, cortisone, tixocortol pivalate, prednisolone, budesonide, pivalone, or methylprednisone.	
Comorbid inflammatory conditions:	
491.x, 492.x, 496.x	Chronic obstructive pulmonary disease
493.x	Asthma
714.x	Rheumatoid arthritis
710.x	Lupus
69x.x	Dermatitis
490.x	Bronchitis
461.x, 473.x	Sinusitis
472.x, 477.x	Rhinitis
725.x	Polymyalgia rheumatic
339.x	Cluster headaches
571.42	Autoimmune hepatitis
IBD-related surgery:	
45.7	Partial excision of large intestine
45.71	Multiple segmental resection of large intestine
45.72	Cecectomy
45.73	Right hemicolectomy
45.74	Resection of transverse colon
45.75	Left hemicolectomy
45.76	Sigmoidectomy
45.79	Other partial excision of large intestine
45.8	Total intra-abdominal colectomy
45.81	Laparoscopic total intra-abdominal colectomy
45.82	Open total intra-abdominal colectomy
45.83	Other and unspecified total intra-abdominal colectomy
45.9	Intestinal anastomosis
45.90	Intestinal anastomosis, not otherwise specified
45.91	Small-to-small intestinal anastomosis
45.92	Anastomosis of small intestine to rectal stump
45.93	Other small-to-large intestinal anastomosis
45.94	large-to-large intestinal anastomosis
45.95	anastomosis to anus (i.e. pouch)
45.6	Other excision of small intestine
45.61	Multiple segmental resection of small intestine
45.62	Other partial resection of small intestine (i.e. jejunectomy, ileectomy, duodenectomy)
45.63	Total removal of small intestine
46.0	Exteriorization of intestine
46.01	Exteriorization of small intestine (i.e loop ileostomy)
46.02	Resection of exteriorized segment of small intestine
46.03	Exteriorization of large intestine
46.04	Resection of exteriorized segment of large intestine
46.1	Colostomy

46.10	Colostomy, not otherwise specified
46.11	Temporary colostomy
46.13	Permanent colostomy
46.14	Delayed opening of colostomy
46.2	Ileostomy
46.20	Ileostomy, not otherwise specified
46.21	Temporary ileostomy
46.22	Continent ileostomy
46.23	Other permanent ileostomy
46.24	Delayed opening of ileostomy
48.40	Pull-through resection of rectum, not otherwise specified
48.42	Laparoscopic pull-through resection of rectum
48.43	Open pull-through resection of rectum
48.49	Other pull-through resection of rectum
48.5	Abdominoperineal resection of rectum
48.50	Abdominoperineal resection of rectum, not otherwise specified
48.51	Laparoscopic abdominoperineal resection of rectum
48.52	Open abdominoperineal resection of rectum
48.59	Other abdominoperineal resection of rectum
48.6	Other resection of rectum
48.61	Trans-sacral rectosigmoidectomy
48.62	Anterior resection of rectum with synchronous colostomy
48.63	Other anterior resection of rectum
48.64	Posterior resection of rectum
48.65	Duhamel resection of rectum
48.69	Other, partial proctectomy
17.31	Laparoscopic multiple segmental resection of large intestine
17.32	Laparoscopic cecectomy
17.33	Laparoscopic right hemicolectomy
17.34	Laparoscopic resection of transverse colon
17.35	Laparoscopic left hemicolectomy
17.36	Laparoscopic sigmoidectomy
17.39	Other laparoscopic partial excision of large intestine

eTable 2. Characteristics of Excluded Patients

Patient-level factors	Overall proportions
n (%)	25,021
Age	
< 50 years	5,343 (21.6%)
51-65 years	8,080 (32.7%)
> 65 years	11,289 (45.7%)
Gender	
Male	23,647 (94.5%)
Female	1,374 (5.5%)
Race	
White	18,684 (74.7%)
Non-white	6,337 (25.3%)
IBD type	
Crohn's disease	9,629 (38.5%)
Ulcerative colitis	14,709 (58.8%)
Indeterminate colitis	683 (2.7%)
Charlson comorbidity index (mean, SD)	
0 to 2	22,359 (89.4%)
Greater than 2	2,662 (10.6%)
Region	
Northeast	4,663 (18.6%)
Southeast	6,466 (25.8%)
Continental	8,545 (34.2%)
Pacific	5,347 (21.4%)
Immunomodulator or biologic use	2,008 (8.0%)
No	23,103 (92.0%)
Outpatient flare	2,291 (9.2%)
No	22,730 (90.8%)
Hospitalization	546 (2.2%)
No	24,475 (97.8%)
Surgery	153 (0.6%)
No	24,868 (99.4%)
Facility-level factors	
Facility complexity	
Highest	9,680 (38.7%)
High	5,152 (20.6%)
Midhigh	4,842 (19.4%)
Medium	2,315 (9.2%)
Low	3,023 (12.1%)
Rural	8,893 (35.5%)
Not rural	16,128 (64.5%)

©2020 Cohen-Mekelburg S et al. *JAMA Network Open*

eTable 3. Sensitivity Analysis of Associations Excluding Deaths

Patient-level factors	Adjusted Odds Ratio (95% Confidence Interval) Excluding deaths
Age	
< 50 years	1.63 (1.50, 1.78)
51-65 years	1.17 (1.09, 1.26)
> 65 years	ref
Gender	
Male	ref
Female	0.95 (0.84, 1.06)
Race	
White	0.96 (0.89, 1.03)
Non-white	ref
IBD type	
Crohn's disease	1.14 (1.07, 1.22)
Ulcerative colitis	ref
Indeterminate colitis	1.15 (1.02, 1.30)
Charlson comorbidity index (mean, SD)	
0 to 2	ref
Greater than 2	1.00 (0.92, 1.09)
Region	
Northeast	0.94 (0.86, 1.04)
Southeast	0.90 (0.82, 0.99)
Continental	0.80 (0.74, 0.88)
Pacific	ref
Baseline corticosteroid-treated flares	0.95 (0.86, 1.05)
No	ref
Baseline IBD-related hospitalizations	1.62 (1.37, 1.91)
No	ref
Baseline Immunomodulator or biologic use	1.42 (1.31, 1.53)
No	ref
Facility-level factors	
Facility complexity	
Highest	ref
High	0.85 (0.79, 0.92)
Midhigh	0.62 (0.57, 0.68)
Medium	0.35 (0.31, 0.40)
Low	0.35 (0.31, 0.40)
Rural	0.99 (0.93, 1.06)
Not rural	ref

©2020 Cohen-Mekelburg S et al. *JAMA Network Open*
